# Medical Treatment Under Community Compulsory Treatment Orders – Are Medications Prescribed Lawfully?

**DOI:** 10.1192/bjo.2025.10601

**Published:** 2025-06-20

**Authors:** Susannah Houston, Louisa Adam, Suzanne Galloway, Martin Carlin, Andrew Donaldson

**Affiliations:** NHS Lanarkshire, Glasgow, United Kingdom

## Abstract

**Aims:** The Mental Welfare Commission (MWC) has provided information outlining good practice for consent to treatment in relation to The Mental Health (Care and Treatment) (Scotland) Act 2003. Patients prescribed psychotropic medications beyond two months of initial detention under the Mental Health Act require a statutory T2 or T3 form, depending on the patient’s ability and willingness to consent to treatment. Without a valid form being in place, there is no legal authority to continue to administer medication.

A MWC report in February 2024 highlighted a number of concerns around the use of community Compulsory Treatment Orders (cCTOs). One of the recommendations from this report was the need to ensure audit processes are in place with regards medical treatment given under cCTOs. This initial audit cycle was commissioned in response to the report and aimed to determine whether T2/T3 forms were (i) in place, (ii) valid and (iii) concordant with what the patient was being prescribed in the community.

**Methods:** Administrative staff from psychiatric subspecialties within NHS Lanarkshire were contacted to gain access to spreadsheets detailing patients detained under a cCTO, as well as online files containing electronic copies of their T2/T3 certificates for review. Patients who were detained under a cCTO at the time of data collection were included (July 2024–October 2024). Review of the patients T2/T3 forms and electronic medical records took place to record whether they were (i) in place, (ii) valid and (iii) reflective of what was currently being prescribed.

**Results:** 89 patients were identified as being on cCTOs. 3 of those did not have T2 or T3 forms in place yet, all for valid reasons.

For the forms that were in place, 96.5% were considered valid though only 65.9% were concordant with what the patient was being prescribed in the community.

**Conclusion:** Almost all patients had appropriate T2/T3 forms in place, suggesting the administrative system in place to manage this is working well.

There were significant discrepancies between what medication had been authorised under T2/3 forms and what the patient was prescribed which will require further review by individual teams.

Recommendations include sharing of the findings, as well as the MWC Good Clinical Practice guidelines, with Responsible Medical Officers and General Practitioners to increase awareness of the need for medication regimes to be compliant with the relevant T2/T3 forms. Additionally, ongoing audit within psychiatry units on an annual basis to improve practice in this area is required to maintain and improve standards.

